# Genetic Diversity, Genetic Structure and Demographic History of the Leaf Beetle *Platycorynus peregrinus* (Herbst, 1783) (Coleoptera: Chrysomelidae) from Thailand

**DOI:** 10.3390/biology14091266

**Published:** 2025-09-13

**Authors:** Satakoon Kaewmungkoon, Nakorn Pradit, Warayutt Pilap, Salakjit Ninlaphay, Takan Chatiwong, Jatupon Saijuntha, Chavanut Jaroenchaiwattanachote, Wittaya Tawong, Warong Suksavate, Pairot Pramual, Chairat Tantrawatpan, Weerachai Saijuntha

**Affiliations:** 1 Mahasarakham Business School, Mahasarakham University, Khamriang Sub-District, Kantharawichai District, Maha Sarakham 44150, Thailand; satakoon.k@msu.ac.th (S.K.); salakjit.n@msu.ac.th (S.N.); tkan.c@msu.ac.th (T.C.); 2 Walai Rukhavej Botanical Research Institute, Mahasarakham University, Maha Sarakham 44150, Thailand; nakorn.p@msu.ac.th (N.P.); warayutt@msu.ac.th (W.P.); 3 Center of Excellence in Biodiversity Research, Mahasarakham University, Maha Sarakham 44150, Thailand; chavanut.j@msu.ac.th; 4 Faculty of Engineering, Mahasarakham University, Maha Sarakham 44150, Thailand; jatupons2534@gmail.com; 5 Department of Agricultural Sciences, Faculty of Agriculture Natural Resources and Environment, Naresuan University, Phitsanulok 65000, Thailand; 6 Center of Excellence in Biodiversity, and Center of Excellence in Research for Agricultural Biotechnology, Naresuan University, Phitsanulok 65000, Thailand; 7 Department of Forest Biology, Faculty of Forestry, Kasetsart University, Bangkok 10900, Thailand; wsuksavate@gmail.com; 8 Department of Biology, Faculty of Science, Mahasarakham University, Kantharawichai District, Mahasarakham 44150, Thailand; pairot.p@msu.ac.th; 9 Division of Cell Biology, Department of Preclinical Sciences, Faculty of Medicine, and Center of Excellence in Stem Cell Research and Innovation, Thammasat University, Rangsit Campus, Pathum Thani 12120, Thailand; 10 Faculty of Medicine, Mahasarakham University, Maha Sarakham 44000, Thailand

**Keywords:** pest, haplotype network, phylogenetic tree, genetic variation, genetic differentiation

## Abstract

*Platycorynus peregrinus* (Herbst, 1783) is a leaf beetle of agricultural importance, yet its genetic diversity and population structure remain poorly understood. We analyzed mitochondrial cytochrome c oxidase subunit I (*COI*) sequences from 147 individuals across 19 populations in Thailand. Forty-five haplotypes were identified, showing high haplotype diversity (0.942) and moderate nucleotide diversity (0.00562). Significant genetic differentiation (Φ_ST_ = 0.0000–0.7857) was strongly associated with geographical distance, and this population structure was further supported by AMOVA (*F*_CT_ = 0.21925, *p* < 0.001). Neutrality tests and mismatch distribution analyses revealed signals of population expansion, alongside evidence of localized differentiation. The substantial genetic variability observed suggests high dispersal ability and possible cryptic lineages. These insights not only advance understanding of the evolutionary dynamics of *P. peregrinus* but also provide valuable guidance for developing sustainable pest management strategies.

## Simple Summary

This study investigates the genetic diversity and genetic structure of the leaf beetle *Platycorynus peregrinus* in Thailand using mitochondrial *COI* gene sequences. Specimens were collected from multiple locations across the country. The results revealed significant genetic diversity both within and among populations, suggesting possible local adaptation and population structure. These findings provide valuable insights into the evolutionary potential of *P. peregrinus* and support the development of more effective pest management and control strategies in the future.

## 1. Introduction

*Platycorynus peregrinus* (Herbst, 1783) is a species of leaf beetle belonging to the family Chrysomelidae, subfamily Eumolpinae. This beetle is primarily phytophagous and secondarily graminivorous, feeding on a variety of host plants [1]. The primary host plants for *P. peregrinus* are members of the genus *Calotropis*, commonly known as the crown flower tree, a toxic shrub characterized by its milky latex. Notably, *P. peregrinus* has been observed visiting more frequently on *Calotropis gigantea*, suggesting a strong preference for species within the *Calotropis* genus [2]. In addition to *Calotropis*, this beetle has also been reported feeding on various species of *Digitaria* (a genus of grasses), indicating a broader host range. Moreover, *P. peregrinus* is known to feed on several agriculturally important crops, including eggplant, okra, tomato, and potato [1]. This dietary versatility suggests that while the beetle has preferred host plants, it is capable of adapting to a variety of plant species, some of which are of economic significance. Both the adult and larval stages of this beetle feed on the leaves of these plants [1].

This leaf beetle is widely distributed in the tropical and subtropical regions, particularly in South and Southeast Asia [2]. Heavy infestations cause severe defoliation, reducing plant vigor and negatively impacting their ecological and economic value [1]. Although this species is agriculturally important, research on its biology and population genetics remains scarce. Knowledge of the genetic diversity of pest species is essential not only for understanding evolutionary processes and population structure, but also for informing pest management strategies [3]. Investigating its genetic variation can provide critical insights into its evolutionary history, population structure, and adaptability to diverse environments. Such information is important for predicting its potential spread and for developing evidence-based management strategies [4,5].

For example, a study on the invasive agricultural pest *Thrips palmi* Karny, 1925 in China demonstrated the importance of population genetic analysis for understanding pest distribution and informing control strategies [6]. Similarly, research on the African fig fly, *Zaprionus indianus* Gupta, 1970, showed that invasive populations can experience reductions in genetic diversity, which may affect their adaptability and fitness [7]. More recently, a study in Thailand reported the genetic variation in the seed bug *Spilostethus pandurus* (Scopoli, 1763), an agriculturally important insect. The results highlighted the species’ adaptability and potential for local differentiation, while the low divergence from populations in other continents suggested ongoing gene flow [8]. Collectively, these studies underscore the crucial role of genetic data in guiding pest management and control efforts.

Mitochondrial DNA (mtDNA) has become a valuable tool in genetic studies, particularly in identifying genetic variation within and between species [9]. The mitochondrial cytochrome c oxidase subunit I (*COI*) gene is widely used as a genetic marker in population genetics and phylogeography due to its relatively high mutation rate and maternal inheritance [10]. *COI* has been extensively employed in the DNA barcoding of species, enabling researchers to distinguish between species, and determining the possibility of cryptic species [11]. Studying the *COI* gene can provide insights into genetic differentiation between geographically isolated populations, reflecting patterns of gene flow, migration, and historical biogeography.

This study aims to examine the genetic diversity, genetic structure and population demographic history of *P. peregrinus* in Thailand by using the mitochondrial *COI* gene as a genetic marker. We seek to assess the genetic variability within populations and determine the degree of genetic differentiation between geographically distinct populations. The findings will contribute to the broader understanding of the population genetics of *P. peregrinus* and provide a baseline for future studies on its evolutionary history. This research will also contribute to the growing body of literature that utilizes mitochondrial markers to assess genetic variation in insect populations, thereby enhancing our understanding of genetic diversity across species and regions.

## 2. Materials and Methods

### 2.1. Sample Collection and Molecular Analysis

**Figure 1:**
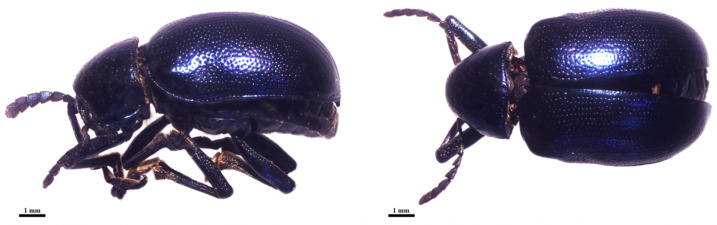
Adult of leaf beetle *Platycorynus peregrinus* in lateral (**left**) and dorsal (**right**) views.

**Figure 2:**
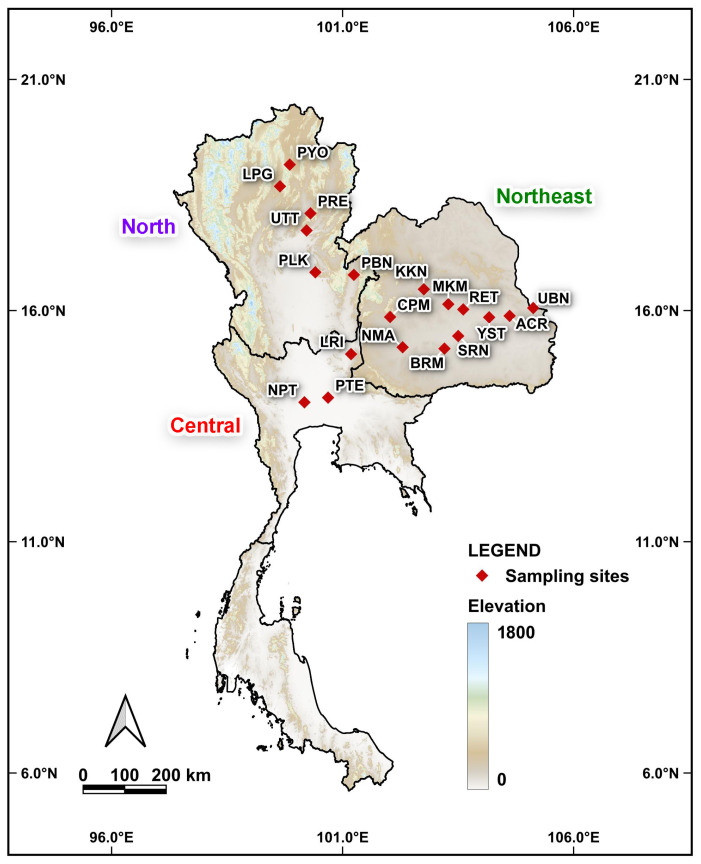
Map showing the sampling localities of *Platycorynus peregrinus* populations across Thailand. Each locality corresponds to the localities listed in Table 1.

We collected 147 individuals of *P. peregrinus* (Figure 1) by hand picking from their host tree, *Calotropis* spp. (crown flower tree) from 19 localities in Thailand (Table 1 and Figure 2). The beetles were immobilized by chilling on ice for approximately 10 min, then immediately transferred to 80% ethanol for preservation. The leaf beetles were randomly selected for species identification by morphology using key to species of the family Chrysomelidae of Thailand reported by [12]. Total DNA was individually extracted from the left foreleg of each leaf beetle using E.Z.N.A.^®^ Tissue DNA kit (Omega bio-tek, Norcross, GA, USA) following the manufacturer’s protocol. The remaining body was preserved as a voucher specimen for morphological reference if needed in the future. DNA samples were kept at −20 °C for further molecular analysis. Partial sequence of the *COI* fragment was amplified and sequenced using primers and PCR conditions as published by [13]. The PCR products were electrophoresed in 1% agarose gels and visualized with GelRed^TM^ Nucleic Acid Gel Stain (Biotium, Inc., Hayward, CA, USA). The amplified band was cut and purified by using E.Z.N.A.^®^ Gel Extraction kit (Omega bio-tek, Norcross, GA, USA). The purified PCR products were sent for DNA sequencing at ATGC Co., Ltd., Thailand.

### 2.2. Data Analysis

All *COI* sequences generated in this study were aligned using the ClustalW program [14] and manually edited in the BioEdit program [15]. Molecular diversity indices and haplotype data were generated using the DnaSp v5 program [16]. A mitochondrial haplotype network was constructed in the Network program version 10.2 (https://www.fluxus-engineering.com/, accessed on 15 July 2025) based on median-joining method [17] using all sequences generated in this study. Neutrality tests (Tajima’s D and Fu’s Fs), genetic differentiation (Φ_ST_) analysis, mismatch distribution analysis, and Analysis of Molecular Variance (AMOVA) were conducted using the Arlequin program version 3.5.2.2 [18]. The resulting Φ_ST_ *p*-values were corrected for multiple comparisons using a Bonferroni correction to avoid type I errors following the formula α_new_ = α/*n*, where α = 0.05 and *n* = 171 is the number of possible pairwise comparisons between the 19 populations. We considered a *p*-value to be statistically significant if it was less than the adjusted threshold of α_new_ = 0.05/171 ≈ 0.00029. Furthermore, the effective population size (N_e_) for each population was estimated, following the formula N_e_ = θ/2μ, where the theta (θ) was estimated in DnaSp v5 (accessed on 15 July 2025) and the mutation rate per site per generation (μ) was assumed to be 1.8 × 10^−8^ [19,20]. To assess the geographical distance separating population groups, an Isolation by Distance (IBD) analysis was conducted to evaluate the correlation between pairwise Euclidean geographical distances and genetic differentiation (Φ_ST_) at the population level. This analysis was performed using the ecodist package [21] in R [22].

### 2.3. Phylogenetic Tree Reconstruction

Phylogenetic trees were constructed using Bayesian inference (BI), Maximum likelihood (ML), and Neighbor joining (NJ) methods. Using the corrected Akaike information criterion (AIC), MrModeltest 2.4 [23] provided the best-fit evolutionary models for the *COI* marker. The GTR + I + G was shown to be the best-fit model for ML and BI phylogenetic analysis. For the BI analysis, we used BEAST X v10.5 [24] with 30,000,000 MCMC iterations, sampling every 1000 iterations. The run was checked for the convergence of the MCMC and effective sample sizes (ESS > 200) in TRACER v.1.7.2 [25]. We used the program Figtree v.1.4.4 [26] to visualize the trees after discarding the first 10% of each MCMC chain as burn-in. The ML and NJ were carried out in MEGA12 [27] with 1000 bootstrap replicates using the GTR + I + G and Kimura 2-parameter (K2P) models, respectively.

## 3. Results

### 3.1. Genetic Variation and Effective Population Size

Mitochondrial *COI* sequences of *P. peregrinus* generated in this study were deposited in GenBank (https://www.ncbi.nlm.nih.gov/genbank/, accessed on 15 July 2025) under accession no. PV400438–PV400482. Across 147 individuals from 19 populations, a total of 45 variable sites were detected, namely 17 singleton variable sites and 28 parsimony informative sites (Table S1). Based on those variations, 45 haplotypes were identified (Pp1–Pp45). Haplotype diversity (Hd) in each population varied between 0 and 0.972 ± 0.064 (average 0.942 ± 0.008). Similarly, the nucleotide diversity (Nd) varies between 0.0000 ± 0.000 and 0.0067 ± 0.0014 (average 0.00562 ± 0.0004) (Table 2). Populations such as PRE, NPT, PBN, LPG, RET, PLK, MKM, and NMA exhibited high haplotype diversity (Hd) (>0.900). Among the 45 haplotypes identified, 32 haplotypes were unique to a single population. PRE and MKM harbored the highest number (4) of unique haplotypes (Uh) (Table 2). Conversely, populations such as KKN, PYO, and UTT had no unique haplotypes, indicating shared haplotypes with other populations.

Estimates of the population mutation parameter (θ) and effective population size (Ne) based on *COI* sequences varied among populations (Table 2). θ ranged from 0.0000 in KKN (no polymorphism) to 0.00811 in PBN, while Ne spanned from 1.49 × 10^5^ (ACR) to 9.01 × 10^5^ (PBN), with an overall mean Ne of 14.0 × 10^5^. Populations such as PBN, PRE, UBN, and NRT showed higher Ne, indicating larger effective sizes and greater genetic variation, whereas ACR, PTE, BRM, and CPM exhibited lower Ne, reflecting reduced diversity and stronger genetic drift. KKN showed Ne = 0, consistent with the absence of variation. These results highlight heterogeneity in genetic diversity, with most populations maintaining large effective sizes, while some display reduced variation likely due to local demographic processes.

### 3.2. Haplotype Network Analysis

**Figure 3:**
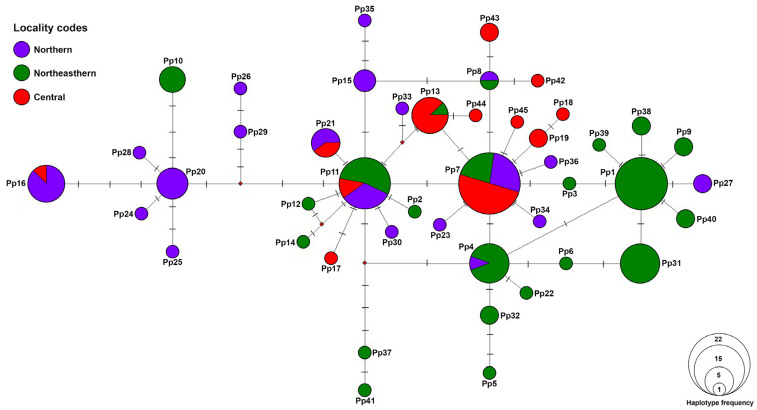
A haplotype network constructed from *COI* haplotypes of *Platycorynus peregrinus* populations in Thailand. Each color represents a different region of sampling localities in Thailand (see Table 1 for details). The size of each circle corresponds to the number of individuals sharing that haplotype. Hash marks on the branches indicate the number of mutational steps between haplotypes.

A mitochondrial DNA haplotype network revealed a complex genetic network among the populations from different geographic regions (Figure 3). A total of 45 haplotypes were identified, with several haplotypes shared across multiple populations and others restricted to specific localities. Three predominant haplotypes (Pp1, Pp7, and Pp11) were the most frequent but only Pp7 and Pp11 are widely distributed while Pp1 was restricted to the northeast of Thailand. Among 45 identified haplotypes, only 7 were shared among geographic regions. The network exhibited a star-like topology, suggesting historical population expansion. Overall, the haplotype network highlights a high level of genetic diversity across populations, with evidence of both historical gene flow and localized differentiation. The distribution of haplotypes suggests a mix of shared ancestral lineages and recent genetic divergence among certain populations.

### 3.3. Phylogenetic Analysis

**Figure 4:**
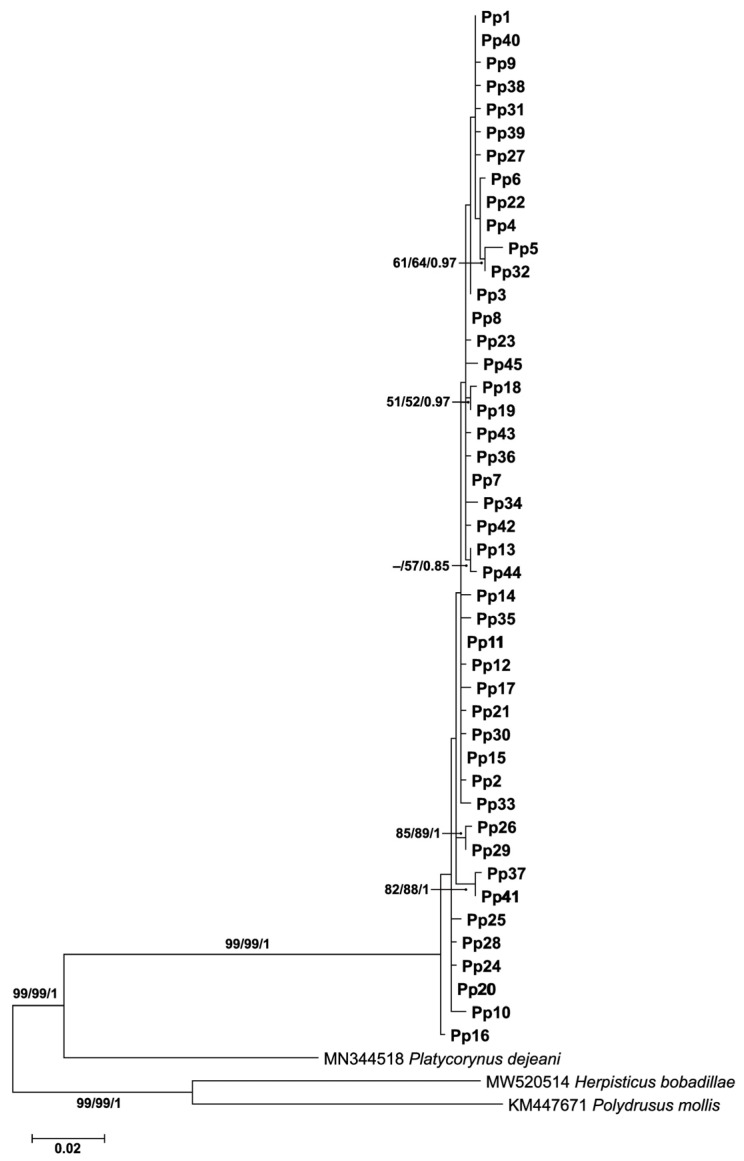
Bayesian phylogenetic tree of 45 *COI* haplotypes (Pp1–Pp45) of *Platycorynus peregrinus* from Thailand. Support values at nodes represent Maximum Likelihood bootstrap, Neighbor-Joining bootstrap, and Bayesian posterior probability, respectively. The en dash (–) indicates supporting values less than 50. Outgroups include *Platycorynus dejeani*, *Herpisticus bobadillae*, and *Polydrusus mollis*.

A Bayesian phylogenetic tree based on *COI* haplotypes was constructed to further examine the genetic relationships among *P. peregrinus* haplotypes (Figure 4). The analysis recovered all 45 haplotypes (Pp1–Pp45) of *P. peregrinus* as a well-supported monophyletic clade, clearly separated from the outgroups (*Platycorynus dejeani* Bertoloni, 1849, *Herpisticus bobadillae*, and *Polydrusus mollis* Ström, 1768). Within *P. peregrinus*, the haplotypes were arranged in shallow branches with generally short internode distances, reflecting low sequence divergence among them. Several small clusters of haplotypes were observed with moderate to high support values (e.g., Pp5 and Pp32, Pp26 and Pp29, Pp41 and Pp37). These results are congruent with the haplotype network analysis (Figure 3), suggesting that *P. peregrinus* represents a single species with high haplotype diversity but shallow genealogical divergence.

### 3.4. Genetic Differentiation and Genetic Structure

**Figure 5:**
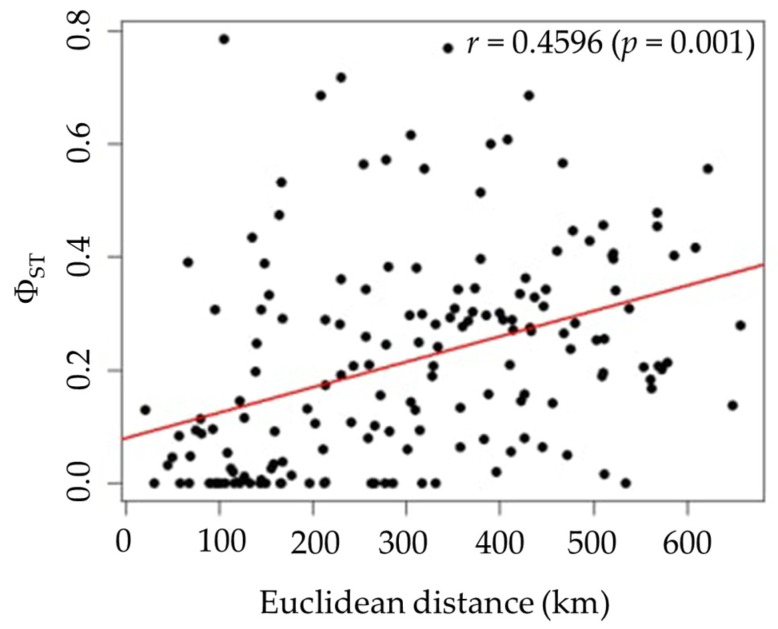
Isolation-by-distance (IBD) by Mantel test based on *COI* sequences, showing the relationship between genetic differentiation (Φ_ST_) and geographic Euclidean distance (km). *r* represents the correlation coefficient, and *p* represents the probability value (*p*-value) assessing statistical significance.

Pairwise Φ_ST_ values among the 19 populations ranged from 0.0000 to 0.7857, indicating a wide range of genetic differentiation (Table 3). While some population pairs showed no significant genetic differentiation, others exhibited significant differentiation, as highlighted by the gray shading Φ_ST_ values in Table 3. The highest genetic difference was observed between CPM and KKN populations (Φ_ST_ = 0.7857, *p* < 0.00001), while consistently low and nonsignificant values were detected among MKM, NMA, SRN, and BRM populations. The table shows clusters of closely related populations with low Φ_ST_ values, while more divergent populations, especially KKN, display consistently higher Φ_ST_ values (Table 3), indicating substantial genetic differentiation. The statistical significance of the Φ_ST_ values is indicated by highlighting the genetic structure across the sampled range. A significant pattern of IBD was detected (Mantel test, *r* = 0.4596, *p* = 0.001), indicating a positive correlation between genetic differentiation (Φ_ST_) and geographic Euclidean distance (Figure 5). This suggests that populations located farther apart are more genetically distinct, consistent with restricted gene flow over increasing distances.

Analysis of Molecular Variance (AMOVA) demonstrated significant genetic differentiation among the three defined groups based on different regions in Thailand, namely northern, central, and northeastern, with *F*_CT_ = 0.21925 (*p* < 0.001). Additionally, significant genetic variation was detected among populations within groups (*F*_SC_ = 0.06520, *p* < 0.05) and among individuals within populations (*F*_ST_ = 0.27016, *p* < 0.001) (Table 4).

### 3.5. Demographic History

**Figure 6:**
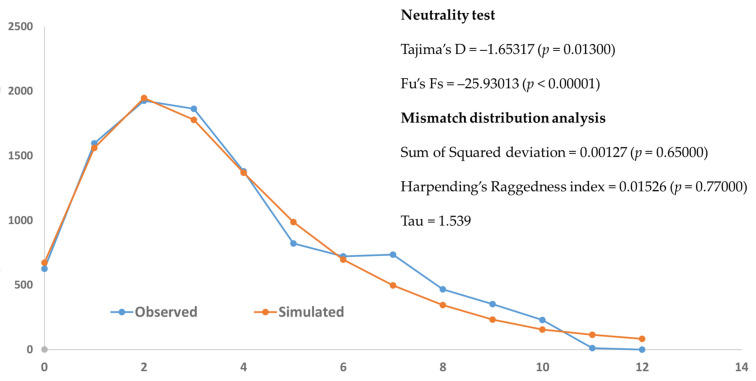
Mismatch distribution graph of *Platycorynus peregrinus* based on *COI* sequence data. The solid blue line represents the observed frequency of pairwise nucleotide differences, while the solid orange line indicates the expected distribution under a model of sudden demographic expansion.

Neutrality tests of all populations yielded significant negative values for both Tajima’s D (–1.65317, *p* = 0.01300) and Fu’s Fs (–25.93013, *p* < 0.00001), suggesting possible population expansion or purifying selection. The mismatch distribution analysis revealed a unimodal and smooth pattern of pairwise differences among sequences, indicating an excess of low-frequency polymorphisms and recent population expansion (Figure 6). The observed distribution did not differ significantly from the expected under sudden expansion model, suggesting that the population has experienced recent demographic expansion. Both sum of square deviation and Harpending’s Raggedness index tests were not statistically significant (Figure 6).

## 4. Discussion

High genetic diversity of *P. peregrinus* was observed in this study, suggesting considerable evolutionary potential that may facilitate adaptation to environmental changes, host plant defenses, and pest control measures. *P. peregrinus* is considered as a pest of various agriculturally important crops, including eggplant, okra, tomato, and potato [1]. A high diversity of plant species can lead to increased genetic variation in herbivorous beetle populations by providing a wider range of food sources and potential habitats, which can drive specialization and genetic differentiation among beetle populations [28]. Therefore, observation of the genetic variation in *P. peregrinus* in this study may indicate the presence of multiple host-adapted lineages. This idea is supported by a previous study on the leaf beetle *Neochlamisus bebbianae* (Brown, 1943), which identified two sympatric host-associated forms, one adapted to maple and the other to willow, and demonstrated that genetic differences related to host use resulted in reduced hybrid fitness, thereby promoting reproductive isolation between host-adapted lineages [29]. In this study, *P. peregrinus* were collected only from crown flower trees; thus, we cannot determine whether the observed genetic differentiation is related to host plant usage or not. Therefore, continued genetic monitoring and host-use studies are essential to better understand host plant usage and genetic differentiation which can be used to support the development of more targeted and effective pest management strategies.

The significant spatial genetic structure observed in the isolation-by-distance (IBD) analysis of *P. peregrinus* populations across Thailand suggests restricted gene flow among geographically separated populations. This pattern may be influenced by geographical distance, dispersal limitations, or natural barriers. Further analysis integrating landscape features and species distribution with genetic differentiation could elucidate their impacts on gene flow. Significant genetic differentiation, as indicated by Φ_ST_ values, reflects barriers to gene flow shaped by ecological or geographic factors. These patterns may be influenced by ecological factors such as host plant availability, climate variability, and habitat fragmentation. Similar patterns have been reported in the willow leaf beetle *Chrysomela aeneicollis* (Schaeffer, 1928), which exhibited significant genetic differentiation among populations correlated with variation in snowpack and temperature [30]. For instance, research on the oak wilt vector beetle *Platypus quercivorus* Murayama, 1925 revealed that populations exhibited genetic structuring influenced by geographical distance and landscape features. This suggests that natural barriers, such as mountain ranges or fragmented habitats, can limit dispersal and gene flow among beetle populations [31]. In Thailand, similar spatial genetic structuring has been reported in the jewel beetle *Sternocera aequisignata* Saunders, 1866 [32], the large brown cricket *Tarbinskiellus portentosus* (Lichtenstein 1796) [13], and the seed bug *S. pandurus* [8].

High genetic diversity might indicate the presence of cryptic species or incipient speciation within the pest population. Recognizing and understanding this cryptic diversity is crucial for effective pest management, as they may differ in behavior, host preference, or susceptibility to control measures. For instance, the whitefly *Bemisia tabaci* Gennadius, 1889 comprises multiple cryptic species with varying reproductive compatibilities and insecticide resistance levels, necessitating tailored management strategies [33]. Similarly, the onion thrips (*Thrips tabaci* Lindeman) consists of distinct lineages exhibiting reproductive isolation and differences in behavior, emphasizing the importance of accurate species identification for effective control [34]. In the context of biological control, the inadvertent introduction of cryptic species can lead to unintended ecological consequences, highlighting the need for thorough genetic assessments prior to agent release [35]. Therefore, integrating molecular tools into pest management programs is essential to detect cryptic diversity, inform targeted control measures, and prevent the misapplication of management strategies.

A primary limitation of this study is the relatively low number of samples per site, which may reduce the resolution of population genetic patterns. In addition, reliance on the *COI* marker alone may limit the detection of finer-scale genetic differentiation, as shown in the paper wasp *Polistes fuscatus* (Fabricius, 1793) [36]. Therefore, future studies incorporating additional nuclear markers could provide more robust insights into population differentiation. We also observed a high frequency of unique haplotypes, suggesting local adaptation to environmental factors such as climate, host plant availability, and predation. Similar habitat-driven genetic differentiation has been reported in other herbivorous insects, such as the seed bug *S. pandurus* [8]. In addition, historical demographic events like bottlenecks, founder effects, and expansions may have influenced the current genetic structure by reducing diversity and later promoting new mutations [37].

We also detected a signal of population expansion in *P. peregrinus* populations in Thailand. However, information on generation time is unavailable, and thus the timing of the population expansion cannot be estimated. Studies in Thailand and Southeast Asia have reported that many insect species have undergone recent population expansion in response to Pleistocene climatic and environmental fluctuations, including the black fly *Simulium chumpornense* Takaoka & Kuvangkadilok, 2000 [38], the fruit fly *Bactrocera latifrons* (Hendel, 1915) [39], *Anopheles* Meigen, 1818 mosquitoes [40,41], and the buffalo fly *Haematobia exigua* Meijere, 1906 [42]. Therefore, we suspect that the population expansion signal observed in *P. peregrinus* may also represent a response of this species to Pleistocene climatic changes.

Lastly, our current finding on genetic variation and population structure of *P. peregrinus* populations in Thailand provides valuable insights not only into their evolutionary dynamics but also into practical pest management. The observed population structure and genetic differentiation suggest that local populations may respond differently to control measures, highlighting the need for region-specific management strategies. Moreover, understanding gene flow among populations can inform the deployment of targeted interventions, such as monitoring high-risk areas for rapid infestations and optimizing the timing of biological or chemical control to limit the spread of genetically distinct populations.

## 5. Conclusions

In conclusion, the level of genetic variation observed within and among populations of *P. peregrinus* in the present study suggests the possible presence of cryptic diversity, which may complicate pest management efforts. The beetle’s dispersal ability and adaptability emphasize the importance of using molecular tools in monitoring and management programs to detect hidden diversity and enhance future control strategies.

## Figures and Tables

**Table 1:** Sampling localities and associated details for *Platycorynus peregrinus* populations collected in Thailand.

Code	*n*	District	Province	Region	Coordinate
LRI	12	Tha Luang	Lop Buri	Central	15.060303° N/101.185584° E
NPT	10	Bang Len	Nakhon Pathom	Central	14.017806° N/100.172494° E
PTE	10	Khlong Luang	Pathum Thani	Central	14.116948° N/100.686501° E
LPG	6	Chae Hom	Lampang	North	18.579162° N/99.627253° E
PBN	10	Lom Sak	Phetchabun	North	16.798181° N/101.252991° E
PLK	9	Wang Thong	Phitsanulok	North	16.826225° N/100.415699° E
PRE	9	Mueang	Phrae	North	18.107199° N/100.317509° E
PYO	5	Mueang	Phayao	North	19.158573° N/99.861056° E
UTT	6	Mueang	Uttaradit	North	17.714014° N/100.223382° E
ACR	6	Mueang	Amnat Charoen	Northeast	15.886554° N/104.616135° E
BRM	10	Satuek	Buri Ram	Northeast	15.269016° N/103.312677° E
CPM	5	Mueang	Chaiyaphum	Northeast	15.857574° N/102.019933° E
KKN	5	Mueang	Khon Kaen	Northeast	16.461561° N/102.768760° E
MKM	9	Mueang	Maha Sarakham	Northeast	16.137739° N/103.294266° E
NMA	7	Non Sung	Nakhon Ratchasima	Northeast	15.202849° N/102.311809° E
RET	6	Mueang	Roi Et	Northeast	16.083239° N/103.570263° E
SRN	10	Chumphon Buri	Surin	Northeast	15.424805° N/103.416461° E
UBN	5	Khemarat	Ubon Ratchathani	Northeast	16.054408° N/105.126661° E
YST	7	Mueang	Yasothon	Northeast	15.859350° N/104.159329° E
Total	147				

*n*, sample size.

**Table 2:** Molecular diversity indices of *Platycorynus peregrinus* from different geographical localities in Thailand, based on *COI* sequences analysis.

Populations	*n*	S	H	Uh	θ	Ne (×10^5^)	Hd ± SD	Nd ± SD
LRI	12	6	5	2	0.00304	3.38	0.788 ± 0.090	0.0025 ± 0.0005
NPT	10	11	7	3	0.00595	6.61	0.933 ± 0.062	0.0045 ± 0.0014
PTE	10	5	5	2	0.00270	3.00	0.800 ± 0.100	0.0020 ± 0.0005
LPG	6	9	5	3	0.00603	6.70	0.933 ± 0.122	0.0059 ± 0.0014
PBN	10	15	8	2	0.00811	9.01	0.933 ± 0.077	0.0057 ± 0.0013
PLK	9	12	6	2	0.00675	7.50	0.917 ± 0.073	0.0066 ± 0.0011
PRE	9	13	8	4	0.00731	8.12	0.972 ± 0.064	0.0066 ± 0.0010
PYO	5	8	4	0	0.00587	6.52	0.900 ± 0.161	0.0067 ± 0.0014
UTT	6	8	3	0	0.00536	5.96	0.800 ± 0.122	0.0065 ± 0.0015
ACR	6	2	3	1	0.00134	1.49	0.600 ± 0.215	0.0013 ± 0.0005
BRM	10	5	5	1	0.00270	3.00	0.867 ± 0.071	0.0032 ± 0.0005
CPM	5	4	4	1	0.00294	3.27	0.900 ± 0.161	0.0028 ± 0.0008
KKN	5	0	1	0	0.00000	n/a	0.000 ± 0.000	0.0000 ± 0.0000
MKM	9	8	7	4	0.00450	5.00	0.917 ± 0.092	0.0037 ± 0.0009
NMA	7	11	5	1	0.00536	5.96	0.905 ± 0.103	0.0060 ± 0.0020
RET	6	8	5	2	0.00540	6.00	0.933 ± 0.122	0.0050 ± 0.0013
SRN	10	10	5	1	0.00734	8.16	0.844 ± 0.080	0.0059 ± 0.0014
UBN	5	10	4	1	0.00499	5.54	0.900 ± 0.161	0.0064 ± 0.0030
YST	7	8	4	2	0.00687	7.63	0.810 ± 0.130	0.0041 ± 0.0015
Total	147	45	45	32	0.01264	14.00	0.942 ± 0.008	0.00562 ± 0.0004

*n*, sample size; S, segregation site; H, number of haplotypes; Uh, unique haplotype; θ, population mutation parameter; Ne, effective population size; Hd, haplotype diversity; Nd, nucleotide diversity; SD, standard deviation; n/a, could not be estimate. Locality codes are provided in Table 1.

**Table 3:** Pairwise genetic differentiation (Φ_ST_) among *Platycorynus peregrinus* populations from 19 different localities in Thailand based on *COI* sequence analysis.

Populations	MKM	NMA	SRN	CPM	UTT	NPT	PYO	ACR	LPG	PRE	BRM	PBN	PLK	RET	UBN	YST	LRI	PTE	KKN
**MKM**	–																		
**NMA**	0.0139	–																	
**SRN**	0.0885	0.0301	–																
**CPM**	0.2476	0.1144	0.0345	–															
**UTT**	**0.3039**	0.1347	0.0798	0.0919	–														
**NPT**	**0.2097**	**0.1016**	0.0781	0.0236	0.0558	–													
**PYO**	**0.4294**	0.2552	0.1683	0.2689	0.1012	0.2019	–												
**ACR**	0.0065	0.0794	0.1986	0.5727	**0.4565**	**0.4067**	0.5571	–											
**LPG**	0.2366	0.0510	0.0000	0.0205	0.0457	0.0164	0.0487	0.4164	–										
**PRE**	**0.2965**	0.1580	0.0642	0.1303	0.0320	0.1421	0.0133	0.4022	0.0189	–									
**BRM**	0.0600	0.0535	0.1293	0.3333	0.3630	**0.2875**	0.4776	0.0267	**0.3092**	**0.3436**	–								
**PBN**	0.1908	0.0599	0.0053	0.0000	0.0484	0.0013	0.0595	**0.3456**	0.0545	0.0145	**0.2458**	–							
**PLK**	**0.2983**	0.1552	0.0642	0.1055	0.1061	0.0942	0.1033	**0.4109**	0.0377	0.0174	**0.3436**	0.0246	–						
**RET**	0.0019	0.0383	0.0939	0.2909	0.3010	**0.2756**	0.3975	0.0211	0.2535	**0.2889**	0.0144	0.2103	0.2937	–					
**UBN**	0.0253	0.0975	0.0353	**0.2424**	0.2059	0.2141	0.2785	0.0468	0.1374	0.1847	0.0034	0.1452	0.1949	0.0423	–				
**YST**	0.0125	0.0000	0.0968	0.2819	0.2645	**0.2370**	**0.4026**	0.0469	0.2081	**0.2824**	0.0269	0.1900	0.2709	0.0104	0.0221	–			
**LRI**	**0.2597**	0.1464	**0.2083**	0.1152	0.2491	0.0913	0.4474	**0.5153**	0.1589	**0.3095**	**0.3605**	0.1320	**0.2897**	**0.3826**	**0.3292**	**0.2821**	–		
**PTE**	**0.2769**	0.1738	**0.2073**	0.1078	**0.2898**	0.0836	**0.4547**	0.5652	0.1897	**0.3131**	**0.3800**	**0.1441**	**0.2974**	**0.3976**	0.3413	**0.3360**	0.0024	–	
**KKN**	**0.3913**	**0.3898**	0.4347	**0.7857**	**0.6151**	**0.5995**	**0.6857**	**0.6857**	**0.6078**	**0.5565**	0.3078	**0.5315**	**0.5639**	0.3076	**0.3438**	**0.4737**	**0.7165**	**0.7683**	–

Bold values indicate statistically significant differences at *p*-value < 0.00029, which was adjusted using Bonferroni correction.

**Table 4:** Analysis of Molecular Variance (AMOVA) based on *COI* sequences of *Platycorynus peregrinus* populations defined by three population groups corresponded to different regions in Thailand, namely northern, central, and northeastern regions.

Source of Variation	d.f.	Ss	Vc	%Va	Fixation Indices
Among groups	2	45.581	0.43940	21.93	*F*_CT_ = 0.21925 **
Among populations within groups	16	35.723	0.10202	5.09	*F*_SC_ = 0.06520 *
Within populations	128	187.22	1.46266	72.98	*F*_ST_ = 0.27016 **

d.f., degree of freedom; Ss, sum of squares; Vc, variance components; %Va, percentage of variation; * *p*-value < 0.05; ** *p*-value < 0.001.

## Data Availability

All data are available upon request.

## Supplementary Materials

The following supporting information can be downloaded at: https://www.mdpi.com/article/10.3390/biology14091266/s1, Table S1: Variable sites in the *COI* sequences among 45 haplotypes of *Platycorynus peregrinus* examined in this study.
